# A new approach of nano-metformin as a protector against radiation-induced cardiac fibrosis and inflammation via CXCL1/TGF-Β pathway

**DOI:** 10.1007/s00210-024-03052-4

**Published:** 2024-04-09

**Authors:** Heba M. Karam, Dina M. Lotfy, Ayman A. Ibrahim, Farag M. Mosallam, Sahar S. Abdelrahman, Amira Abd-ElRaouf

**Affiliations:** 1 Drug Radiation Research Department, National Center for Radiation Research and Technology, Egyptian Atomic Energy Authority, Cairo, Egypt; 2 Department of Chemistry and Chemical Biology, McMaster University, 1280 Main Street West, Hamilton, ON L8S4L8 Canada; 3 Anatomic Pathology Department, Faculty of Veterinary medicine, Cairo University, Cairo, Egypt

**Keywords:** Nano-metformin, Gamma radiation, CXCL1, Troponin, TGF, NF-κB

## Abstract

The present work investigates the potential role of metformin nanoparticles (MTF-NPs) as a radio-protector against cardiac fibrosis and inflammation induced by gamma radiation via CXCL1/TGF-β pathway. Lethal dose fifty of nano-metformin was determined in mice, then 21 rats (male albino) were equally divided into three groups: normal control (G1), irradiated control (G2), and MTF-NPs + IRR (G3). The possible protective effect of MTF-NPs is illustrated via decreasing cardiac contents of troponin, C-X-C motif Ligand 1 (CXCL1), tumor growth factor β (TGF-β), protein kinase B (AKT), and nuclear factor-κB (NF-κB). Also, the positive effect of MTF-NPs on insulin-like growth factor (IGF) and platelet-derived growth factor (PDGF) in heart tissues using immunohistochemical technique is illustrated in the present study. Histopathological examination emphasizes the biochemical findings. The current investigation suggests that MTF-NPs might be considered as a potent novel treatment for the management of cardiac fibrosis and inflammation in patients who receive radiotherapy or workers who may be exposed to gamma radiation.

**Graphical Abstract:**

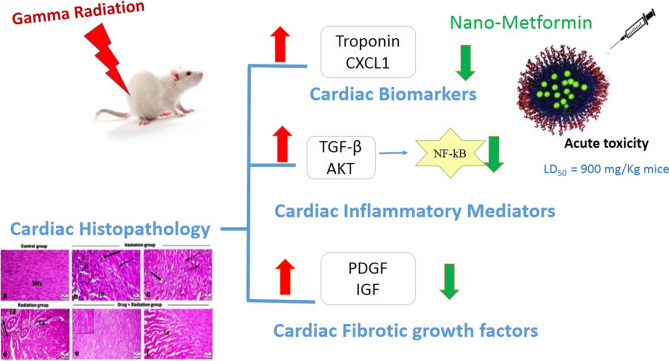

## Introduction

Nowadays, with the multiple exposures to radiation in both diagnostic and therapeutic fields, there is a parallel growing urge to study and develop radio-protective therapies that can help in the betterment of the overall outcome of radiation usage in medical fields. Toxicities that are concomitant to radiotherapy are either short term, which commonly heal within weeks to months, or long term, generally considered irreversible or progressive. Ionizing radiation is known to produce direct and indirect double-strand breaking of the DNA, which is the main mechanism of inducing cell death in cancer radiotherapy. Yet, normal neighboring tissue might act differently where the sensitivity to radiation and the doses received differ from that in cancer cells. Chest radiotherapy for treatment of lung adenocarcinomas, Hodgkin’s and non-Hodgkin’s lymphomas, breast cancers, and Wilms tumors generates a number of cancer survivors that are subject to developing cardiovascular diseases (Gujral et al. 2016, Zheng et al. 2017). The most common cardiotoxicity manifestation induced by radiotherapy is fibrosis, which is a common denominator in a number of cardiac diseases like myocardial infarction, cardiomyopathy, arrythmia, and hypertensive heart disease. In response to radiation, acute inflammatory action produced by pro-fibrotic growth factors takes place (e.g., TGF-β, PDGF, bFGF, and IGF) leading to vasodilation and vascular permeability. This acute phase may be followed by further upregulation of fibrotic and pro-inflammatory cascades leading to chronic disease (Taunk et al. 2015; Spetz et al. 2020).

Metformin (or dimethylbiguanid), on the other hand, is a well-established blood glucose–lowering drug. It is ranked as the first-choice drug for management of diabetes (type II) because of its high efficacy and its potentiality in preventing progressive impaired glucose tolerance (prediabetic), as well as treatment of gestational diabetes (Bailey 2017). With the privilege of its high safety profile, many studies have experimented repurposing of metformin, and the results were very promising, where metformin was found to have an anti-cancer effect through preventing growth of different types of tumors (Skuli et al. 2022) as well as through interfering with key immunological cascades that are associated with tumorigenesis (Ma et al. 2020). Metformin was also found to have an anti-aging action, either through direct effect on different pathways involved in cell aging (Torres et al. 2020) or indirectly through improving health span of diabetic and cardiovascular disease patients (Mohammed et al. 2021). Furthermore, metformin has a distinguished neuroprotective action (Nandini et al. 2019, Paudel et al. 2020) and cardioprotective effect (Driver et al. 2018, Karam and Radwan 2019).

Silver metformin nanoparticles (MTF-NPs) have shown superior efficiency compared to metformin in vitro (Abbas et al. 2022), in addition to its modified pharmacokinetic parameters and slower elimination rate (Pereira et al. 2021). In the context, the present work sheds the light on the prospect cardioprotective effect of MTF-NPs against radiation-induced cardiotoxicity, and the potentiality of using it as a co-treatment for radiotherapy patients. To evaluate this effect, an acute toxicity in vivo study was performed for silver metformin nanoparticles in mice. Non-toxic dose was chosen to illustrate the positive effect against gamma irradiation–caused cardiac toxicity in rats, through evaluating cardiac enzyme troponin-I which is considered to be the biomarker of choice in acute cardiac syndromes due to its early release directly after cardiac cell injury (Garg et al. 2017), as well as evaluating this damage through CXCL/TGF-β pathway. Both inflammatory and pro-fibrotic pathways were examined to compare the extent of acute damage with and without the metformin dose through evaluation of NF-κB and AKT, as well as growth factors (PDGF and IGF). In addition, for confirmation, histopathological examinations were done.

## Materials and methods

### Material

#### Animals

Male mice (20–25 g) and adult albino Wistar rats (130–150 g) were obtained from Egyptian Atomic Energy Authority, Cairo, Egypt. Animals were kept randomly in groups inside cages (28 × 43 × 18 cm) for 7 days in the laboratory. Animals were allowed free access to food consisting of standard pellets chow (El-Nasr Chemical Co., Cairo, Egypt) and water ad libitum.

Animals’ handlings were performed according to the guidelines of Animal Ethics Committee of the Egyptian Atomic Energy Authority, Cairo, Egypt (28A/23).

#### Treatment

##### Metformin nanoparticle preparation

Metformin nanoparticle preparation was provided by the microbiology laboratory at Drug Radiation Research Department, Egyptian Atomic Energy Authority, Cairo. Egypt. The drug was prepared according to Abbas et al.’s method (Abbas et al. 2022).

##### Radiation process

Whole-body gamma radiation exposure of animals was done by radiation source ^137^Cs using Canadian Gamma Cell (GC-40; Nordion-Canada) at a dose rate of 0.43 Gy/min to deliver 6 Gy. This source belongs to the Egyptian Atomic Energy Authority, Cairo, Egypt. The cell was calibrated by alanine dosimetry relative to a primary standard.

### Methods

#### Estimation of acute toxicity (lethal dose fifty)

Lethal dose fifty (LD_50_) of MTF-NPs was investigated in albino mice according to the method of Chinedu et al. (2013). Since the LD_50_ of the MTF-NPs was carried out using mice, the equivalent dose was calculated for rats using the table of Paget and Barnes (1964).

#### Design of the main study

Twenty-one male albino rats were used in this study and distributed equally as follows: Group I rats received deionized water (normal control). Group II rats were exposed to 6 Gy single dose of gamma radiation (irradiated group). Group III rats received 900 mg/kg/day i.p. (1/10 LD_50_) of MTF-NPs, daily for 2 weeks, then 2 h after the last dose they were exposed to 6 Gy gamma radiation (MTF-NPs + irradiation group).

Rats were sacrificed under deep anesthesia using urethane (1.2 mg/kg) (Flecknell 1993) 24 h after the irradiation process. Heart tissues were dissected out and rinsed with saline then dried on filter paper, and divided into two parts. The sides were kept in 10% formalin for histological and immunological studies and the left parts were homogenized in ice-cold 0.1 M phosphate buffer saline (pH 7.4) and stored at −80°C for biochemical examinations.

#### Biochemical examinations of troponin, CXCL1, TGF, AKT-β, and NF-κB in heart homogenates using ELISA techniques

Troponin (catalogue number: SL0121Mo), CXCL1 (catalogue number: E-EL-R0003), AKT (catalogue number: EK720692), TGF-β (catalogue number: 670.070.128), and NF-κB (catalogue number: E-EL-R0611) contents were determined using their specific antibodies by ELISA techniques in line with the manufacturer’s instructions, and anti β-actin (Thermo Fisher Scientific, Rockford, Illinois, USA, 1:1000) served as an internal control.

#### Immunohistochemical analysis of IGF and PDGF in heart tissues

Expression of PDGF and IGF were detected immunohistochemically using avidin–biotin peroxidase (DAB; Sigma Chemical Co.) on paraffin slices of hearts from the control and all treated groups according to the method described by El-Daly et al. (2023). Monoclonal antibodies for PDGF and IGF (1:200 and 1:100 dilutions, respectively) (Abcam, Cambridge, USA) were used to be incubated with tissue slices as well as the reagents required for the avidin–biotin peroxidase (Vectastain ABC peroxidase kit; Vector Laboratories) method for the detection of the antigen–antibody complex. The chromagen 3,3-diaminobenzidine tetrahydrochloride was used to visualize each marker’s expression (DAB; Sigma Chemical Co.). Using image analysis software, the positive brown region of each marker’s expression was quantified as an optical density in 7 high-power microscopic fields (ImageJ, 1.46a; NIH, USA).

#### Histopathological examination of heart tissues

Heart tissue specimens of rats of various groups were fixed for 24 h in 10% buffered neutral formalin before routinely processed into paraffin sections. The specimens were cleaned in distilled water, dehydrated in ethanol dilutes, and clarified in xylene. Finally, paraffin blocks were prepared and chopped into 4- to 5-μm-thick portions. The tissue slices were mounted on glass slides, then slices were deparaffinized and stained with hematoxylin and eosin (H&E) according to the method of Suvarna et al. (2013). All histopathology investigations were conducted by a professional investigator who was blinded throughout the sample identification process to avoid bias.

### Statistical analysis

Data were expressed as means ± SEM. Comparisons between means were done using one-way analysis of variance (ANOVA) test followed by Tukey–Kramer multiple comparisons test at *p* <0.05. However, for histopathological scoring, results were analyzed using the Kruskal–Wallis ANOVA test. GraphPad Prism software package version 6 (GraphPad Software Inc., USA) was used for calculation of all statistical tests.

## Results

### Toxicity study

The acute toxicity of MTF-NPs was evaluated by estimation of its lethal dose of 50% (LD_50_) in albino male mice. Dose was 900 mg/kg (i.p.) by weight. Consequently, 1/10 of the dose was chosen as the effective dose for the investigation of the efficacy of MTF-NPs as cardiac protector.

### Effect of MTF-NPs on heart parameters of rats exposed to gamma radiation

**Fig. 1:**
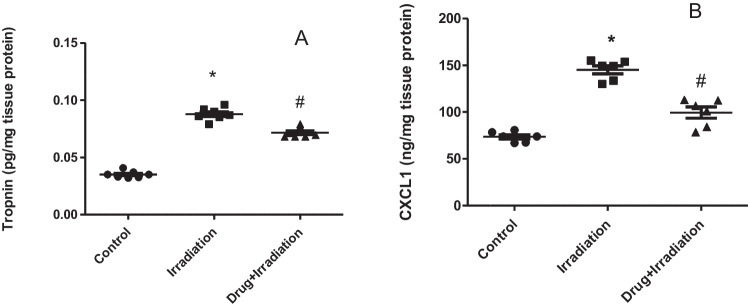
Effects of metformin nanoparticles on cardiotoxicity induced in rats exposed to gamma radiation: **A** Troponin; **B** CXCL1. Each value represents mean ± SEM. Statistical analysis was carried out by one-way ANOVA followed by Tukey–Kramer multiple comparisons test (*n* = 7). *Significantly different from normal control. ^#^Significantly different from irradiated control at *p* < 0.05

Heart damages were induced by exposure to gamma radiation as shown by the significant increase in the activities of both troponin (148.6%) and CXCL1 (92%), respectively, compared to normal rats, while administration of metformin nanoparticles orally normalized the previous ratios (Fig. 1A,B).

### Effect of metformin nanoparticles on heart inflammatory parameters in rats exposed to gamma radiation

The current study illustrated that gamma radiation exposure induced inflammation as shown by the increase in TGF-β, NF-κB, and AKT contents by 99.6%, 58.2%, and 26.5%, respectively.

**Fig. 2:**
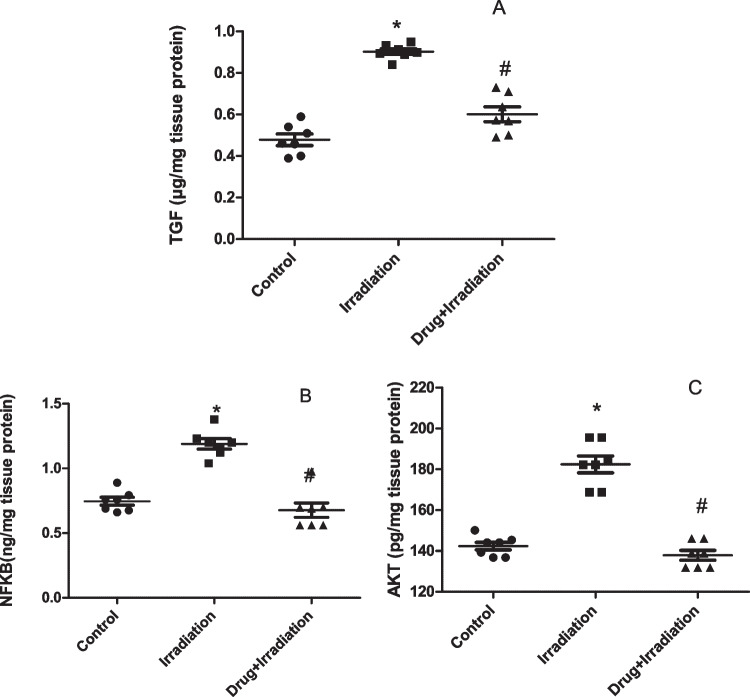
Effect of metformin nanoparticles on cardiac inflammation induced in rats exposed to gamma radiation: **A** TGF-β, **B** NF-κB, and **C** AKT. Each value represents mean ± SEM. Statistical analysis was carried out by one-way ANOVA followed by Tukey–Kramer multiple comparisons test (*n* = 7). *Significantly different from normal control. ^#^Significantly different from irradiated control at *p* < 0.05

On the other hand, oral administration of metformin nanoparticles decreased the rise which occurred in the previous parameters by 90 and 44%, respectively, compared to irradiated rats (Fig. 2A–C).

### Effect of metformin nanoparticles on (immunohistochemistry) in gamma-irradiated rats

**Fig. 3:**
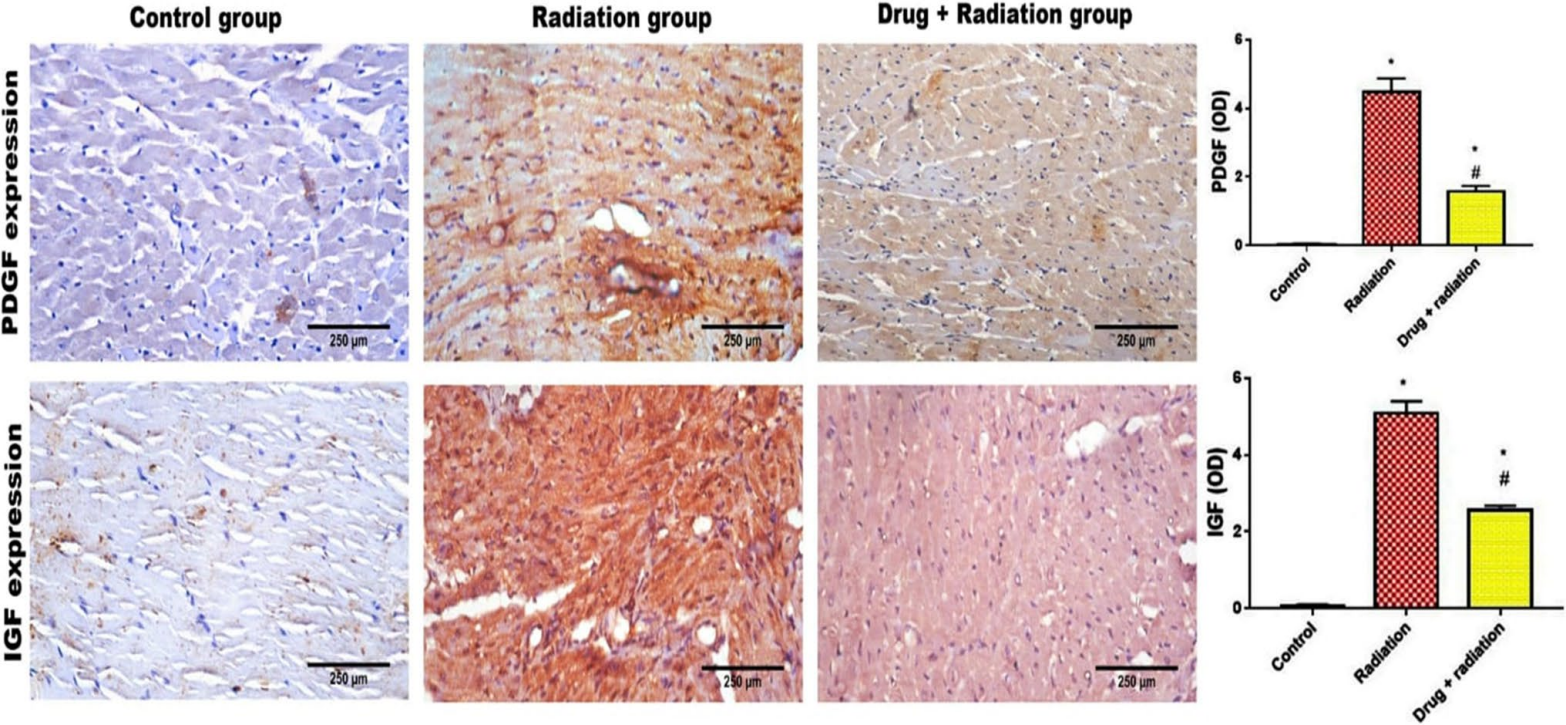
Effect of metformin nanoparticles on cardiotoxicity-induced alterations in pro-fibrotic growth factors in rats exposed to gamma radiation: PDGF and IGF. Each value represents mean ± SEM. Statistical analysis was carried out by one-way ANOVA followed by Tukey–Kramer multiple comparisons test (*n* = 7). *Significantly different from normal control. ^#^Significantly different from irradiated control at *p* < 0.05

It is clearly noticed that treatment of rats with the N-Met before being irradiated could clearly amend the increased expression of both PDGF and IGF (Fig. 3A,B). Hearts’ sections of control rats as well as metformin nanoparticle–treated rats show negative immune expression of both PDGF and IGF while the irradiated group showed a significant increase in the expression of both markers. Irradiated rats that were pre-treated with N-Met show a significant decrease in the expression of both markers in their cardiac muscle fibers, illustrated as positive brown color of each marker.

### Histopathological results

**Fig. 4:**
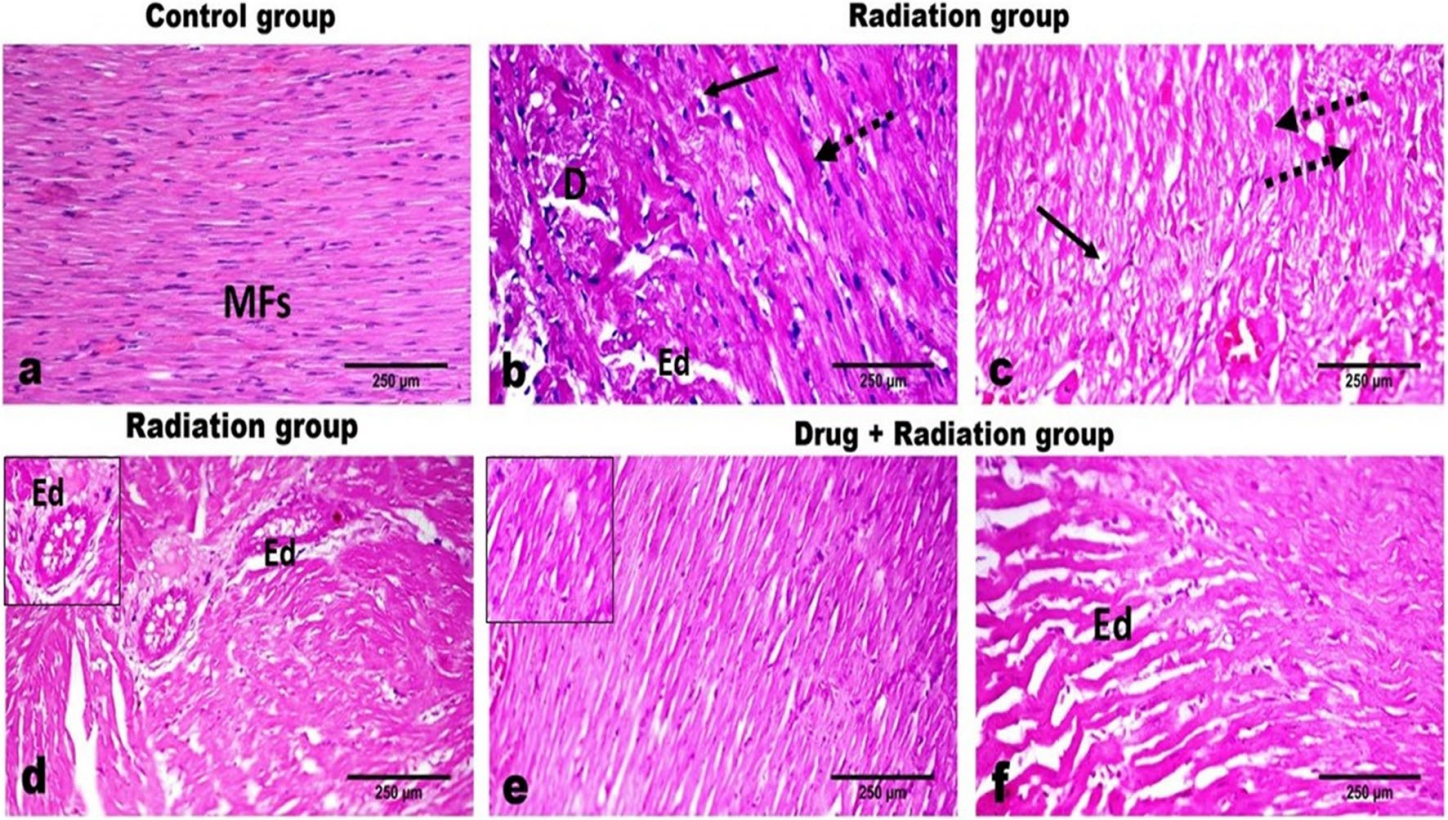
Effect of metformin nanoparticles on cardiotoxicity-induced alterations in histopathological examination: **a** control group; **b**–**d** radiation group; **e**, **f** N-Met pre-treated radiation group

Heart sections of control rats showed normal cardiac muscle fibers with cross striations (Fig. 4a) and normal coronary blood vessel branches while hearts of irradiation-exposed rats showed diffusely cardiac muscle sarcoplasmic swelling, degeneration, and loss of striation. Multifocal pallor, disorganization, intermuscular edema, and fragmentation were commonly observed (Fig. 4b). The degenerate cardiomyocytes have swollen vacuolated sarcoplasm with loss of cross-striation and pyknotic nuclei (Fig. 4c). Frequent necrotic cardiomyocytes appear darkly eosinophilic, hyalinized with or without pyknotic nuclei. The coronary vessels in the vicinity showed hypertrophied endothelial linings, mild medial thickening vacuolation, and marked perivascular edema with sometimes mild perivasculitis that is characterized by mild lymphoplasmacytic infiltration perivascularly (Fig. 4d).

**Fig. 5:**
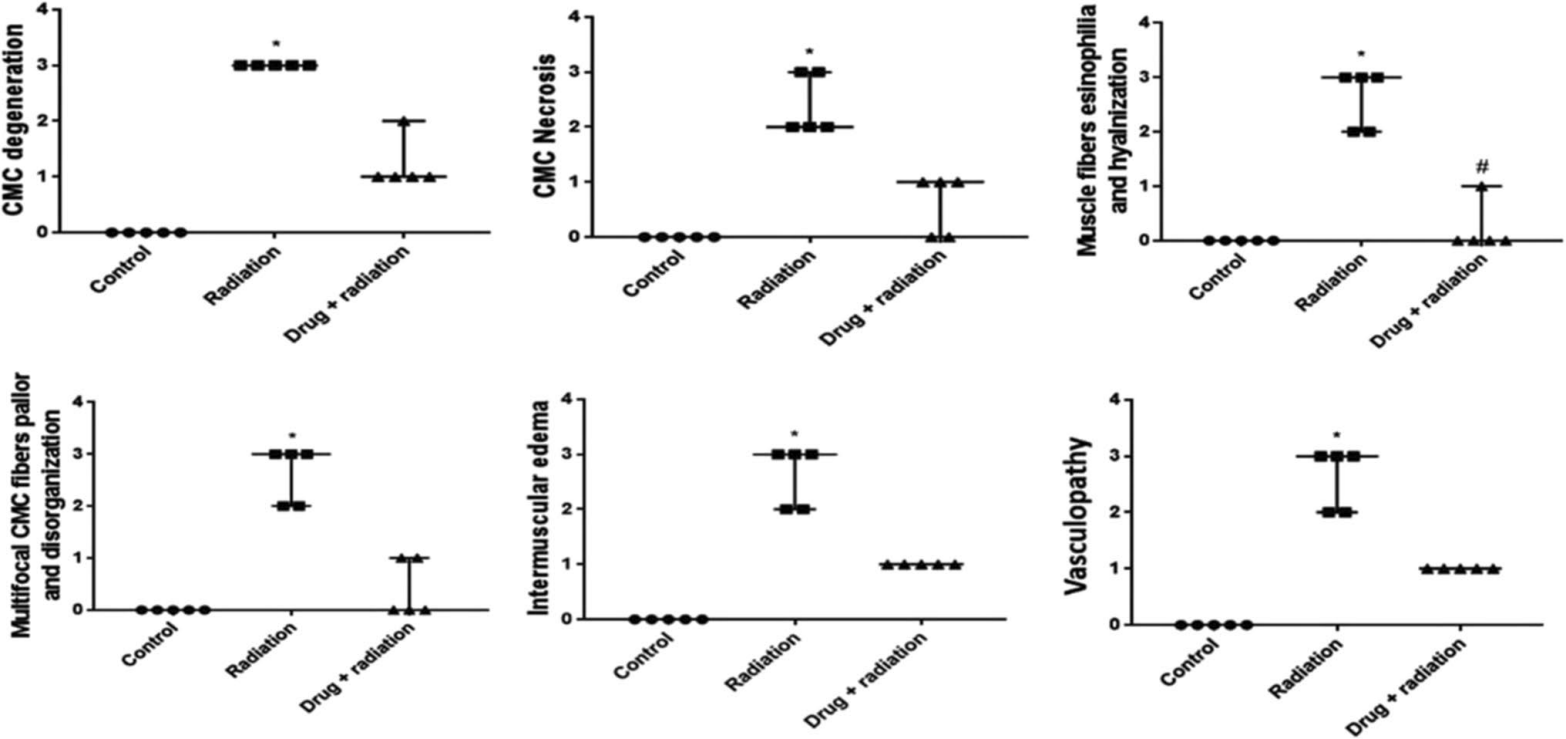
Effect of metformin nanoparticles on cardiotoxicity-induced alterations in scoring of histopathological results. Each value represents median ± SEM. Statistical analysis was carried out by one-way ANOVA followed by Kruskal–Wallis multiple comparison test. *Significantly different from normal control. ^#^Significantly different from irradiated control at *p* < 0.005

On the other hand, hearts of irradiated rats that were pre-treated with metformin nanoparticles showed mild vascular congestion and mild degeneration of the cardiomyocytes (Fig. 4e). Few focal areas of disorganization of myocardiocytes with mild intermuscular edema can be noticed (Fig. 4f). The scoring of the severity of the observed histopathological changes is presented in Fig. 5.

## Discussion

Ionizing radiation (IR) is known to have both direct and indirect damaging effects through double-strand breaking (DSB) of the DNA and the release of reactive oxygen species (ROS), respectively (Ravanat and Douki 2016, Helm and Rudel 2020). Moreover, its effect on normal tissue is characterized by bystander effect and adverse tissue events. Adverse tissue events are effects of irradiation on the non-cancer tissue that are attributed to mechanisms other than cell death (Foray et al. 2016). Radiotherapy-induced cardiovascular toxicity is one of the most known side reactions of chest radiotherapy, leading to a spectrum of cardiac disorders including arrhythmia, cardiac abnormalities, valvular disease, ischemic heart disease, and cardiomyopathy (Azimzadeh et al. 2011, Boerma et al. 2016, Koutroumpakis et al. 2020). The release of cytokines after radiation exposure is time dependent, peaking usually at 4–24 h after radiation exposure (Di Maggio et al. 2015).

Troponin is a cardiac protein that is found in the cytoplasm of myocytes, and together with Ca^+2^ content they regulate the interaction between actin and myosin filaments, producing the cardiac muscle contraction (Tahir et al. 2019). The subunit Tn-I is exclusive to the cardiac muscles, where studies failed to identify it in other organs. Thus, the occurrence of Tn-I in the blood stream is clear evidence of myocardial injury. Findings of the present study had shown a significant elevation (148.6%) in the content of Tn-I in the serum of rats in the irradiated groups, which indicates the acute myocardial damage induced by the 6-Gy dose of irradiation. CXCL as well has elevated significantly (92%) by radiation reflecting active inflammatory response. CXCL is a member in pro-inflammatory cytokine C-X-C motif family. CXCL has a moderating role in recruitment and redirecting of neutrophils and monocytes to the injured tissues of myocardium and arterial wall in cases of cardiovascular disease and heart failure (Park et al. 2013, Wang et al. 2018). CXCL together with metalloproteinases MMP and caspases are target genes of IL-17A, which is produced in infarcted myocardial regions, and leads to fibrosis and apoptosis (Pordel et al. 2018).

On the other hand, pre-treatment with metformin nanoparticles reduced the levels of both Tn-I and CXCL1 significantly, probably through its anti-inflammatory activity (Dehkordi et al. 2018; Cameron et al. 2016) and cardioprotective effect (Efentakis et al. 2019, Ajzashokouhi et al. 2020). Cardioprotection by metformin was observed through a significant decrease in Tn-I level in rats after chemotherapeutic cardiac damage (Aruna and Gayathiri 2018, El Kiki et al. 2020). Metformin has shown a modulatory effect on IL17 and CXCL1 in keratinocyte cell culture, and this effect was justified by its interference with I-1β and affecting all its consecutive inflammatory cascades (Tsuji et al. 2020).

CXCL exerts its activity by binding to its receptor CXCR2 mainly expressed on neutrophils, and this activation of CXCL/CXCR2 axis diverges into many pathways which help in cardiac damage. Recruitment of neutrophils and leukocytes participates in atherosclerosis and cardiac fibrosis (Wang et al. 2018), as well as activation of TGF-Smad2/3 signaling pathway that induces cardiac fibrosis (Zhang et al. 2020). TGF-β is an active player in heart fibrosis in both experimental models and clinical studies through inducing Smad-dependent pathway (Zhao et al. 2008), as well as promoting α-SMA transcription in fibroblasts (Dobaczewski et al. 2010, Jiang et al. 2021). TGF together with angiotensin-II and PDGF and other inflammatory cytokines enhance the differentiation of cardiac fibroblasts into myofibroblasts, leading to fibrosis (Leask 2015). In the current study, irradiation elevated TGF-β (99.6%) showing initial fibrotic activity as well as elevation of both NF-kB (58.2%) and AKT (26.5%). AKT plays a critical role in cardiovascular function where it interferes with cell survival pathways, growth and proliferation, angiogenesis, vasorelaxation, and myocyte metabolism (Abeyrathna and Su 2015). It is also linked to cardiomyocyte hypertrophy (Wu et al. 2021).

Pre-treatment by metformin nanoparticles succeeded to amend the increased expression of inflammatory mediators including TGF-β, NF-kB, and AKT to a normal level (statistically not significant from control group); this result suggests that the anti-inflammatory effect of metformin might be through direct effect on CXCL1, thus downregulating all the subsequent inflammatory and fibrotic cascades.

Furthermore, immunohistological expression of both PDGF and IGF was also affected by irradiation and modulated by metformin nanoparticle pre-treatment. There is an interactive relationship linking the activity of both TGF-β and PDGF in different organs (Trojanowska 2008) (Porsch et al. 2014) where PDGF plays an important role in proliferation and migration, in addition to recruitment of fibroblasts, monocytes, and myocytes, as well as increasing synthesis of collagen (Czarkowska-Paczek et al. 2006). Zhao et al. explored the TGF-β pathway and PDGF fibroblast regulatory effect and collagen turnover through treating cardiac fibroblast by PDGF, and they concluded that PDGF-D significantly enhanced TGF-β1 synthesis and significantly elevated proliferation of cardiac fibroblast (Zhao et al. 2013).

Similarly, IGF also promotes synthesis of collagen by fibroblasts leading to its deposition in the heart muscle. In addition, IGF regulates cardiac metabolism and growth leading to hypertrophy, and increases the secretion of Tn-I, α-actin, and myosin light chain-2 (Castellano et al. 2009). Results of our histopathological examinations have confirmed the protective action of metformin nanoparticle drug, where lower scoring and almost normalized results were observed with respect to degeneration, necrosis, edema, and myocyte disorganization.

## Conclusions

All together with these results, MTF-NPs could be a promising treatment as a protector against radiation-induced cardiac fibrosis and inflammation.

## Data Availability

Data supporting the investigations of the present study are available within the manuscript.
