# Correction to: A new approach of nano-metformin as a protector against radiation-induced cardiac fibrosis and inflammation via CXCL1/TGF-Β pathway

**DOI:** 10.1007/s00210-024-03194-5

**Published:** 2024-06-01

**Authors:** Heba M. Karam, Dina M. Lotfy, Ayman A. Ibrahim, Farag M. Mosallam, Sahar S. Abdelrahman, Amira Abd-ElRaouf

**Affiliations:** 1 Drug Radiation Research Department, National Center for Radiation Research and Technology, Egyptian Atomic Energy Authority, Cairo, Egypt; 2 Department of Chemistry and Chemical Biology, McMaster University, 1280 Main Street West, Hamilton, ON L8S4L8 Canada; 3 Anatomic Pathology Department, Faculty of Veterinary medicine, Cairo University, Cairo, Egypt

**Correction to: Naunyn-Schmiedeberg's Archives of Pharmacology**

10.1007/s00210-024-03052-4

The published original article, contained a mistake in one of the author names.

The name of Dr. Ayman M. Ibrahim should be corrected as “Ayman A. Ibrahim”.

In addition, ORCID details are added to Dena Lotfy, Amira Abd-Elraouf, Ayman A. Ibrahim

The original article has been corrected.

